# Effect of Pulse Duration and Direction on Plasticity Induced by 5 Hz Repetitive Transcranial Magnetic Stimulation in Correlation With Neuronal Depolarization

**DOI:** 10.3389/fnins.2021.773792

**Published:** 2021-11-26

**Authors:** Islam Halawa, Katharina Reichert, Aman S. Aberra, Martin Sommer, Angel V. Peterchev, Walter Paulus

**Affiliations:** ^1^Department of Clinical Neurophysiology, University Medical Center Göttingen, Göttingen, Germany; ^2^Medical Research Division, National Research Center, Cairo, Egypt; ^3^Department of Biomedical Engineering, Duke University, Durham, NC, United States; ^4^Department of Neurology, University Medical Center Göttingen, Göttingen, Germany; ^5^Department of Geriatrics, University Medical Center Göttingen, Göttingen, Germany; ^6^Department of Psychiatry and Behavioral Sciences, Duke University, Durham, NC, United States; ^7^Department of Electrical and Computer Engineering, Duke University, Durham, NC, United States; ^8^Department of Neurosurgery, Duke University, Durham, NC, United States; ^9^Department of Neurology, Ludwig-Maximilians University of Munich, Munich, Germany

**Keywords:** rTMS (repetitive transcranial magnetic stimulation), pulse duration and energy, direction of stimulation, rTMS intensity, MEPs

## Abstract

**Introduction:** High frequency repetitive transcranial magnetic stimulation applied to the motor cortex causes an increase in the amplitude of motor evoked potentials (MEPs) that persists after stimulation. Here, we focus on the aftereffects generated by high frequency controllable pulse TMS (cTMS) with different directions, intensities, and pulse durations.

**Objectives:** To investigate the influence of pulse duration, direction, and amplitude in correlation to induced depolarization on the excitatory plastic aftereffects of 5 Hz repetitive transcranial magnetic stimulation (rTMS) using bidirectional cTMS pulses.

**Methods:** We stimulated the hand motor cortex with 5 Hz rTMS applying 1,200 bidirectional pulses with the main component durations of 80, 100, and 120 μs using a controllable pulse stimulator TMS (cTMS). Fourteen healthy subjects were investigated in nine sessions with 80% resting motor threshold (RMT) for posterior-anterior (PA) and 80 and 90% RMT anterior-posterior (AP) induced current direction. We used a model approximating neuronal membranes as a linear first order low-pass filter to estimate the strength–duration time constant and to simulate the membrane polarization produced by each waveform.

**Results:** PA and AP 5 Hz rTMS at 80% RMT produced no significant excitation. An exploratory analysis indicated that 90% RMT AP stimulation with 100 and 120 μs pulses but not 80 μs pulses led to significant excitation. We found a positive correlation between the plastic outcome of each session and the simulated peak neural membrane depolarization for time constants >100 μs. This correlation was strongest for neural elements that are depolarized by the main phase of the AP pulse, suggesting the effects were dependent on pulse direction.

**Conclusions:** Among the tested conditions, only 5 Hz rTMS with higher intensity and wider pulses appeared to produce excitatory aftereffects. This correlated with the greater depolarization of neural elements with time constants slower than the directly activated neural elements responsible for producing the motor output (e.g., somatic or dendritic membrane).

**Significance:** Higher intensities and wider pulses seem to be more efficient in inducing excitation. If confirmed, this observation could lead to better results in future clinical studies performed with wider pulses.

## Introduction

The therapeutic use of repetitive transcranial magnetic stimulation (rTMS) has been shown to have level A efficacy in the treatment of depression and chronic pain (Lefaucheur et al., 2020). The main mechanism of its action is thought to be induction of synaptic plasticity producing either long term potentiation (LTP) or long term depression (LTD) (Huerta and Volpe, 2009; Vlachos et al., 2017). This is supported by the fact that responses to rTMS exhibit some properties of Hebbian synaptic plasticity (Hebb, 1949) depending on the different stimulation parameters, such as stimulation frequency and intensity (Bliss and Cooke, 2011; Pell et al., 2011).

The effects of key rTMS parameters, i.e., stimulation frequency, intensity, and number of pulses and sessions, on plastic aftereffects have been closely investigated (Rossini et al., 2015). However, there are few studies on the impact of pulse duration on rTMS outcome due to the scarcity of devices with adjustable pulse durations (Peterchev et al., 2011).

The controllable pulse parameter TMS device (cTMS3, Rogue Research Inc., Montreal, QC, Canada) allows varying the duration of its near-rectangular pulses using two capacitors and four transistors that alternate the current between the capacitors (Peterchev et al., 2014). The ratio of capacitor voltages is defined as the *M*-ratio, which determines the relative amplitudes of the different phases of the pulse waveform. Using *M* = 0.2 (lower values more unidirectional), the pulse duration of a single TMS pulse was recently found to bias the balance of excitation and inhibition (Hannah et al., 2020). We have already shown that with inhibitory 1 Hz rTMS (*M* = 0.2) only the widest pulse duration (120 μs) switched the 1 Hz inhibitory aftereffects to a significant increase in excitability, while 40 and 80 μs pulses produced the expected inhibition (Halawa et al., 2019).

In an early LTP experiment using high frequency stimulation of rat cortices and treating pulse duration as a surrogate for intensity, stimulation with wider pulses led to significant neuronal potentiation (McNaughton et al., 1978). The authors argued that increasing intensity and pulse durations stimulated more afferent inputs, which, in turn, enhanced their cooperativity and induced greater potentiation of synaptic transmission.

Here, we used cTMS to test the effect of increasing pulse durations on the aftereffects of 5 Hz rTMS, a protocol known to induce excitatory aftereffects (Ziemann et al., 2008; Rossi et al., 2009; Rothkegel et al., 2010). To interpret the effects of coil orientation, pulse duration, and pulse intensity on the neuromodulatory effects, we also simulated the relative membrane polarization induced by each pulse using a first-order linear model.

## Materials and Methods

### Participants

As in our prior study, which showed a significant effect of pulse duration on neuromodulation with 1 Hz rTMS (Halawa et al., 2019), we recruited 15 subjects for this study. Of these, 14 subjects (4 men and 10 women with a mean age of 23.5 ± 2.6 SD years) completed the study. This sample would allow us to detect an effect size of *f* = 0.29 for alpha power of 0.05, beta power of 0.90, and the study design involving 9 within-subject measurements (estimated with G*Power). All participants were right-handed (Oldfield, 1971), free from any neurological or psychiatric disorders, taking no centrally acting medications, and had no contraindications to TMS (Rossi et al., 2009). A resting motor threshold (RMT) of more than 70% MSO for a Magstim 200^2^ device was an exclusion criterion to prevent overheating the cTMS coil delivering the rTMS, particularly for the wider pulses.

We obtained written informed consent from each subject before participation. The local ethics committee of the University Medical Center Göttingen approved the study protocol, which conformed to the Declaration of Helsinki.

### Recordings

Motor evoked potentials (MEPs) were recorded from the first dorsal interosseous (FDI) muscle of the right hand with surface Ag–AgCl electrodes in a belly-tendon montage. The electromyography signals were amplified, band-pass filtered (2 Hz–2 kHz), and digitized at a sampling rate of 5 kHz with a micro-1401 AD converter (Cambridge Electronic Design Ltd., Cambridge, United Kingdom). All signals were stored digitally for offline analysis. The peak-to-peak MEP amplitude served as an index for M1 excitability. The participants were requested to relax the right FDI during the measurements. Individual traces contaminated by voluntary muscle contraction before the MEP response were excluded from analysis.

### Transcranial Magnetic Stimulation

For the pre- and post-rTMS MEP measurements, TMS was delivered over the M1 representation with a Magstim 200^2^ (Magstim Co. Ltd., Whitland, United Kingdom). The 5-Hz rTMS protocol was delivered via a cTMS3 device.

**FIGURE 1:**
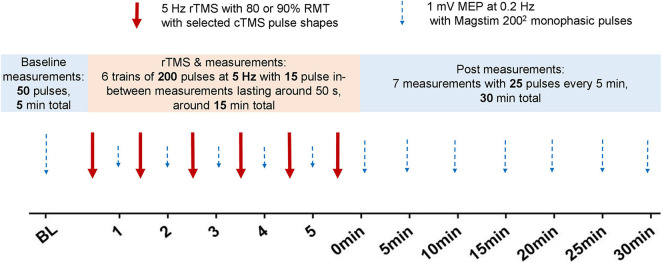
Timeline of the experimental sessions. The sessions were randomized, and there was at least a 1-week pause between sessions to avoid possible carry-over effects.

Repeated, randomized sessions were performed, six for the 80% RMT anterior–posterior (AP) and posterior–anterior (PA) and three for the 90% RMT AP condition. They were separated by at least one week to avoid carry-over. Each session consisted of three steps as shown in Figure 1.

Step 1: Determining thresholds and baseline:

For each session, we used the Magstim 200^2^ with the D70 coil to determine the RMT and the MSO intensity that gave a response of approximately 1 mV for the baseline measurement intensity in the PA direction with monophasic pulses. In addition, we determined the RMT for the cTMS pulse shape used in the interventions, both for the PA and the AP direction as a reference for the 5-Hz rTMS stimulation. The induced current direction was reversed by electronically switching the current pulse direction with the coil position fixed. The baseline measurements consisted of a 50-pulse series at 0.2 Hz using the previously determined MSO intensity that gave a 1-mV response in the PA direction with monophasic pulses.

**FIGURE 2:**
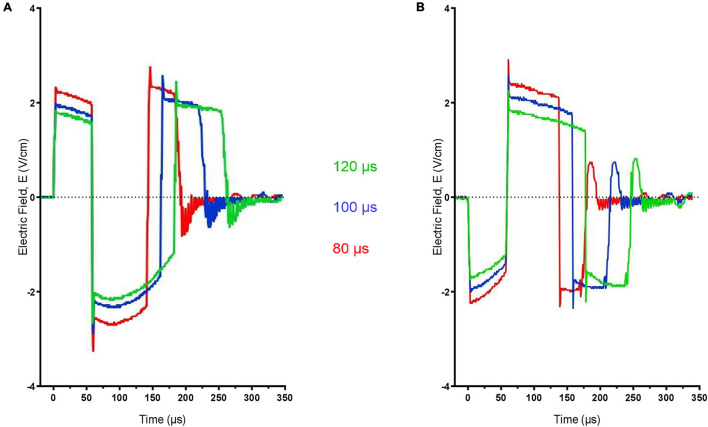
Waveform and electric field strength of the repetitive transcranial magnetic stimulation (rTMS) pulse shapes recorded directly at the center 2 cm away from the TMS coil at the average threshold intensities for 5 Hz posterior–anterior (PA) stimulation **(A)** and 5 Hz anterior-posterior (AP) stimulation **(B)**.

Step 2: Interventional cTMS stimulation:

We used customized, bidirectional pulse shapes which could be generated at 5 Hz with no decay or variation of intensity, which we verified using an external electric field (E-field) probe coil (Koponen et al., 2020). We stimulated at 5 Hz using three durations of the second electric field phase, i.e., 80, 100, and 120 μs in the PA and the AP directions at 80% RMT and in the AP direction at 90%; the first phase was fixed at 60 μs (Figure 2).

In accordance with Rothkegel et al. (2010), we applied 1,200 pulses in six 200-pulse blocks separated by 15 MEP measurements at 0.2 Hz using the same intensity that produced 1 mV at baseline from the Magstim 200^2^ device (the interval between the blocks thus lasted approximately 50 s).

Step 3: After the final rTMS pulse block, we applied 25 pulses at 0.2 Hz using the 1 mV baseline intensity and repeated the series every 5 min for 30 min using the Magstim 200^2^ stimulator.

### Statistical Analysis

For RMT, a two-way repeated measures ANOVA was used with the within-subject factors direction (PA and AP) and pulse duration (80, 100, and 120 μs) for the 80% PA and AP conditions. We conducted another repeated measures ANOVA for the AP condition with the within subject factors intensity (80 and 90%) and pulse duration to explore inter-session stability of RMT. For MEP amplitude changes, we first ran two one-way ANOVAs with the within-subject factor condition (across all direction, intensity, and pulse duration combinations) for the average of the 50 baseline MEP measurements and stimulation intensity needed to elicit 1 mV MEP, which were used for the baseline and post measurements, to exclude baseline differences between sessions. Then, for each session, MEP amplitudes for each subject were normalized by dividing the averaged in-between and post measurement values by the average of the subject’s baseline measurement. Subsequent MEP measurements were binned together for the in-between, 0–15 min and 20–30 min measurements within each subject. A repeated measures ANOVA was used to test for the effect of the within-subject factors condition (PA 80%, AP 80%, and AP 90%), pulse duration (80, 100, and 120 μs), and time (baseline, in-between, 0–15 min, and 20–30 min) on the normalized MEP amplitude as a dependent variable. Sphericity violation was tested for by Mauchly’s test and corrected for by Greenhouse-Geisser method if violated. Given the exploratory nature of the study, *post hoc t*-tests were conducted comparing the MEP amplitude change to the baseline for different pulse durations even though the pulse duration factor was not found significant in affecting MEP amplitudes in the ANOVA. Correction for multiple comparisons was done with the Bonferroni-Dunn method.

### Neural Membrane Polarization Model

We recorded the cTMS pulse waveforms using the E-field probe coil sampled at a rate of 1 MHz. We then used a first-order low pass filter with time constant τ_*m*_ (Barker et al., 1991; Corthout et al., 2001; Peterchev et al., 2013) to approximate membrane polarization induced by each pulse waveform. We used this model to estimate both the strength–duration time constant from experimental motor threshold measurements (Peterchev et al., 2013; D’Ostilio et al., 2016; Aberra et al., 2020) as well as the peak depolarization for a range of time constants, representing different neuronal elements in cortex (cell bodies, axons, dendrites). For the former case, we estimated separate time constants for the PA and AP RMT measurements (D’Ostilio et al., 2016) and compared them using paired two-tailed *t*-tests. For the latter case, we extracted the peak depolarization for each pulse scaled by the mean stimulation intensity across subjects applied in each rTMS protocol, i.e., 80% RMT for the PA pulses and 80 and 90% RMT for the AP pulses. Since the linear membrane model has no spatial dimension or explicit direction, we tested two different definitions of pulse waveform polarity: (1) PA-directed E-field produced depolarization and AP-directed E-field produced hyperpolarization (“PA depolarizing”) or (2) AP-directed E-field produced depolarization and PA-directed E-field produced hyperpolarization (“AP depolarizing”). These two definitions represent neural elements with opposite directions relative to the E-field. We then performed least-squares, linear regression for the peak depolarization and average normalized MEP amplitudes for both polarity definitions.

## Results

### RMT

**FIGURE 3:**
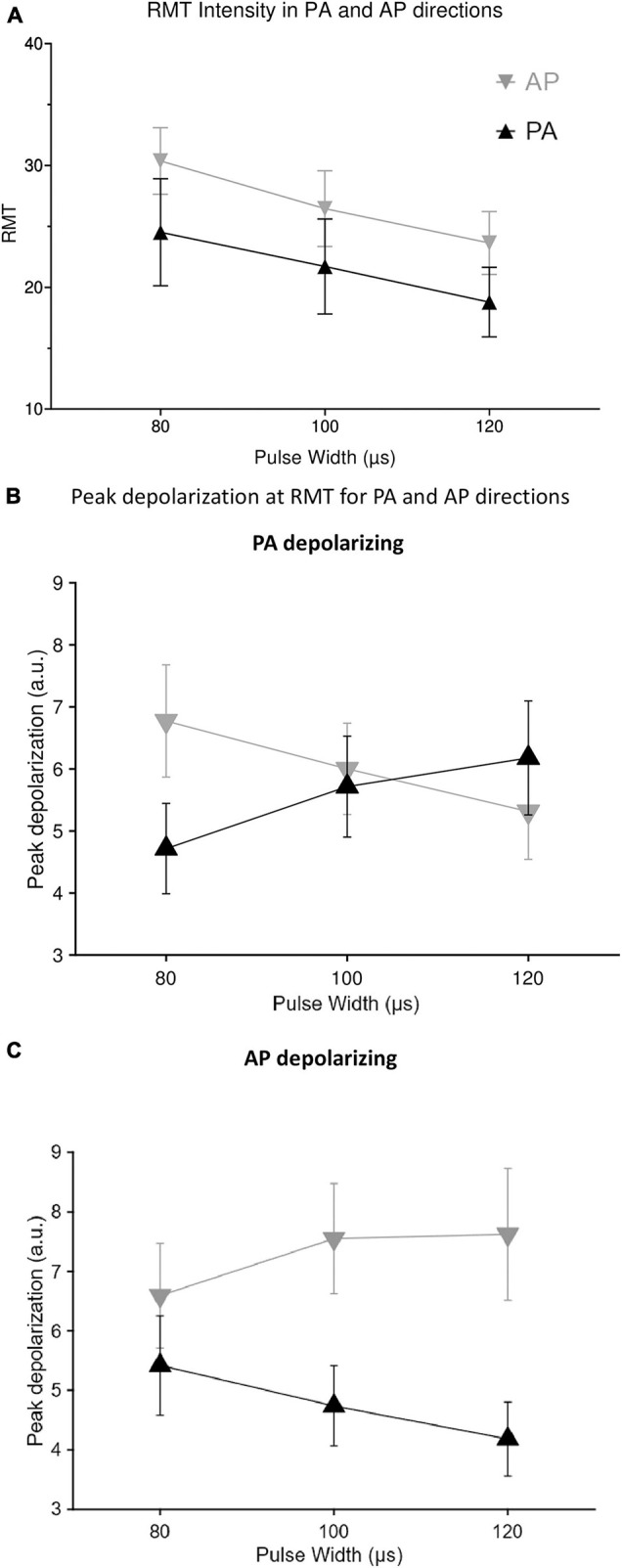
Effect of pulse duration and stimulation direction on **(A)** intensity threshold and resulting depolarization for **(B)** posterior-anterior (PA) direction defined as depolarizing and **(C)** anterior-posterior (AP) direction defined as depolarizing.

For the direction conditions, PA 80% and AP 80% there was a significant effect of direction [*F*_(1, 13)_ = 71.2743, *p* < 0.0001] and pulse duration [*F*_(2, 26)_ = 99.374, *p* < 0.0001] on RMT, but the interaction was not significant [*F*_(2, 26)_ = 0.209, *p* = 0.813] with wider pulses resulting in significantly lower RMTs (Figure 3A). There was no significant variability in RMT values across the two intensity conditions of AP stimulation [*F*_(1, 13)_ = 0.398, *p* = 0.539]. Direction dependent depolarization exhibited different patterns across pulse durations, relevant to the direction of the main phase. The longer AP pulses induced more depolarization in the AP direction but less in the PA direction (Figure 3B), while the longer PA pulses induced more depolarization in the PA direction but less in the AP direction (Figure 3C). The strength–duration time constant estimated separately for each individual was 71.57 ± 24.31 μs (mean ± SD) for the PA direction and 107.3 ± 29.81 μs for the AP direction (*p* < 0.01). Similarly, estimating a single group time constant and individual rheobase values gave a time constant of 67.85 μs [CI = (57.91 μs, 77.80 μs)] for PA and 102.13 μs [89.67 μs, 114.6 μs] for AP.

### Plastic Aftereffects

Baseline one-way ANOVA revealed no significant difference on MEP amplitude across sessions [*F*_(8, 117)_ = 0.4619, *p* = 0.8806]. Intensity used in measuring the baseline and subsequent measurements was not significantly different across sessions [*F*_(8, 117)_ = 0.06611, *p* = 0.9988].

Repeated measures ANOVA for the three conditions 80% PA, 80% AP, and 90% AP revealed significant main effects of condition [*F*_(2, 26)_ = 6.011, *p* = 0.007], and time [*F*_(3, 39)_ = 4.645, *p* = 0.007], and a significant interaction between these factors [*F*_(6, 78)_ = 3.126, *p* = 0.008]. The main effect of pulse duration was not significant [*F*_(2, 26)_ = 0.052, *p* = 0.949] as well as its interaction with condition [*F*_(4, 52)_ = 0.33, *p* = 0.857], time [*F*_(6, 78)_ = 0.127, *p* = 0.993], and condition with time [*F*_(12, 156)_ = 0.475, *p* = 0.927]. Therefore, *post hoc* tests of the effect of pulse duration were performed on an exploratory basis.

**FIGURE 4:**
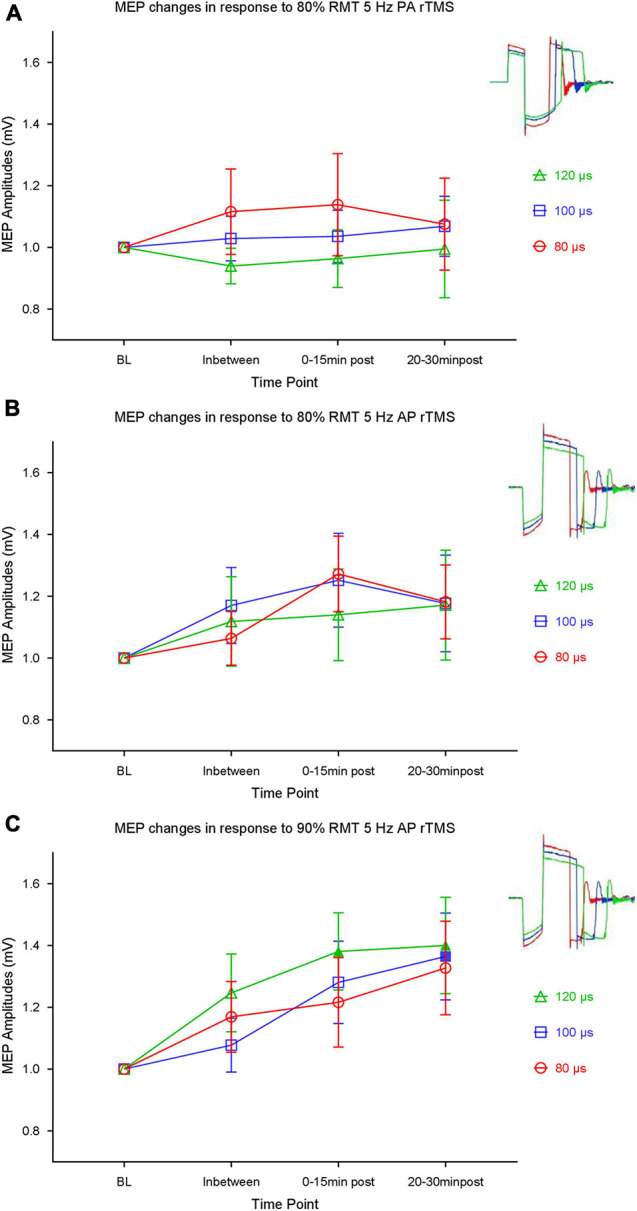
Mean and SEM of motor evoked potential (MEP) amplitude changes in response to: **(A)** 80% resting motor threshold (RMT) 5 Hz stimulation using 80, 100, and 120 μs main component in the posterior-anterior (PA) direction. **(B)** 80% RMT 5 Hz stimulation using 80, 100, and 120 μs main component in the anterior-posterior (AP) direction. **(C)** 90% RMT 5 Hz stimulation using 80, 100, and 120 μs main components in the AP direction. Pulse shapes used for stimulation are illustrated in corresponding colors in the top right corner.

For the 80% RMT, PA-directed 5 Hz rTMS, *post hoc t*-tests for the time bins during and after intervention with the baseline revealed no significant shift from the baseline (Figure 4A). Again, with the 80% RMT AP-directed stimulation, *post hoc t*-tests for the time bins with the baseline revealed no significant shift from the baseline (Figure 4B).

For the 90% AP stimulation conditions, *post hoc t*-tests of the in-between and the post bins showed that the 120 μs pulse shape produced significant excitation at the two post time bins: 0–15 min bin (Bonferroni adjusted *p* = 0.0156) and 20–30 min bin Bonferroni (adjusted *p* = 0.0490), as compared to baseline. The 100 μs condition produced excitation only at the 20–30 min bin (Bonferroni adjusted *p* = 0.0461). Eighty microseconds stimulation did not induce significant shift of excitability from the baseline at any time point and no significant difference was found between pulse durations (Figure 4C).

**FIGURE 5:**
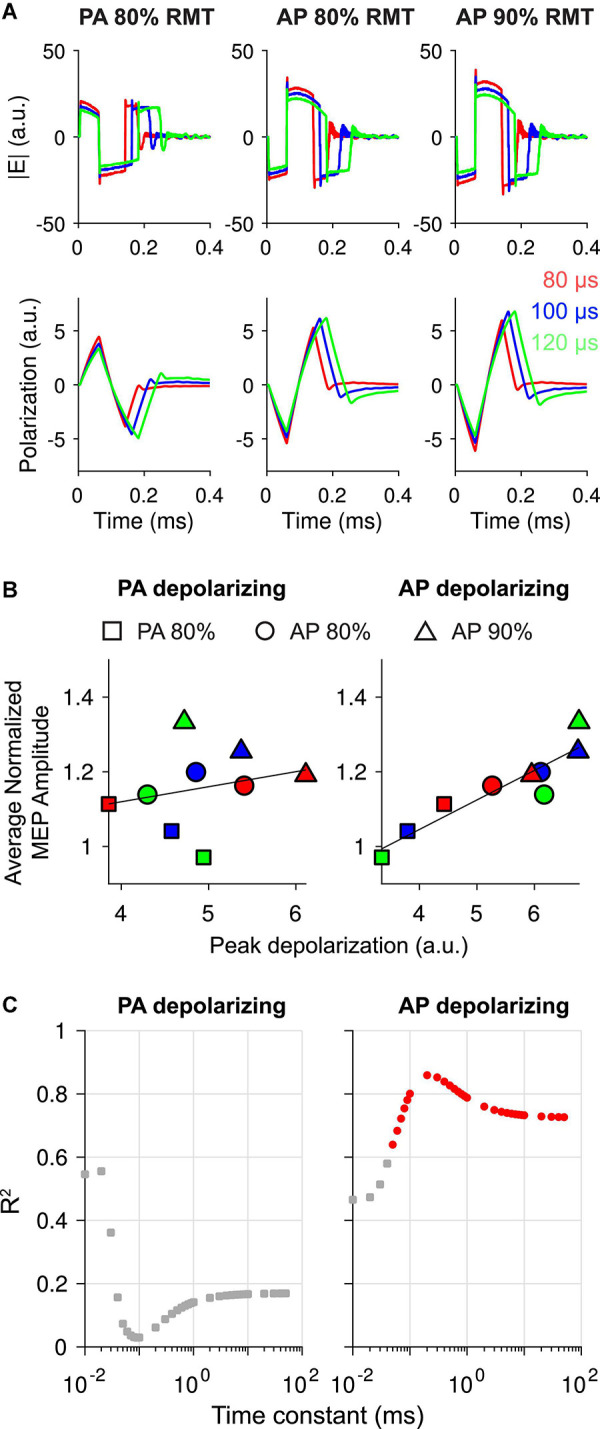
**(A)** E-field waveforms scaled to mean intensity applied in experiments (top row) and simulated polarization for linear membrane model with 200 μs time constant (bottom row). **(B)** Correlation between average normalized motor evoked potential (MEP) amplitudes and peak depolarization values in panel **(A)**, with linear regression overlaid, for the main phase of posterior-anterior (PA) defined as depolarizing (left) or anterior-posterior (AP) defined as depolarizing (right). **(C)** R^2^ values for linear regression of average normalized MEP amplitudes and peak depolarization as a function of membrane time constant. Red circles indicate *p* < 0.01, while gray squares indicate *p* > 0.01. PA depolarizing on left and AP depolarizing on right.

Finally, we simulated the polarization induced by each pulse scaled to the mean intensity applied experimentally across subjects and extracted the peak depolarization for each stimulation condition for both polarity assumptions (Figure 5A). Focusing first on a single time constant of 200 μs based on previous measurements with different cTMS pulses (Peterchev et al., 2013; D’Ostilio et al., 2016), the average normalized MEP amplitude was linearly correlated with the peak depolarization when AP E-field was defined as depolarizing in its main phase (R^2^ = 0.86; *p* < 0.001), but not when PA was defined as depolarizing (R^2^ = 0.06; *p* = 0.520) (Figure 5B). When the AP E-field direction was defined as depolarizing, the PA pulses were hyperpolarizing in their main phase. For the PA pulses, peak depolarization was therefore produced by the initial, reversed phase, which was 60 μs for all pulse waveforms. This produced an inverse correlation between main pulse duration and peak depolarization for PA pulses, as RMT, and consequently applied pulse intensity, decreased with pulse duration. As a result, the trend in peak depolarization relative to pulse duration matched the trend in mean MEP modulation. We found strongest correlations between the AP depolarizing linear membrane model and experimental data with the 200 μs time constant, which corresponds to neural elements with higher time constants than the strength–duration time constant estimated from the motor threshold data (57.91–114.58 μs) (Table 1). Furthermore, the correlation coefficient was significantly reduced for shorter time constants (*R*^2^ = 0.47; *p* = 0.043 for τ_*m*_ = 10 μ*s*) and, to a lesser extent, for longer time constants, eventually reaching an asymptotic level (*R*^2^ = 0.73; *p* = 0.004 for τ_*m*_ = 50 *ms*) (Figure 5C, right). In contrast, we did not find statistically significant correlations for the opposite polarity definition (PA depolarizing) for any membrane time constant (Figure 5C, left).

**TABLE 1:** Strength–duration curve parameters estimated using measurements of resting motor threshold (RMT) with bidirectional cTMS3 pulses with main (2nd) E-field phase of 80, 100, and 120 μs duration applied in the posterior-anterior (PA) and anterior-posterior (AP) direction.

Model	Parameters	PA		AP	
		Mean	SD or 95% CI	Mean	SD or 95% CI
Individual rheobase and group time constant	Rheobase (% MSO)	13.82	1.97	13.10	1.66
	Time constant (μs)	67.85	[57.91, 77.80]	102.13	[89.67, 114.58]
Individual rheobase and individual time constant	Rheobase (% MSO)	13.72	3.08	13.10	3.46
	Time constant (μs)	71.58	24.31	107.27	29.81

## Discussion

This study suggests that increasing both the pulse duration and intensity can increase 5 Hz rTMS excitatory aftereffects. An exploratory finding was that with the higher intensity of 90% RMT, 5 Hz rTMS in the AP direction increased cortical excitability relative to baseline for pulses with dominant E-field phase duration of 120 and 100 μs, whereas the 80 μs pulses did not. Hence, the effect of increasing the pulse duration is similar to what is already known for increasing stimulation intensity (Modugno et al., 2001; Fitzgerald et al., 2006). The difference between the 80 and 90% RMT AP conditions suggests that a certain degree of direct activation or depolarization, proportional to pulse intensity, is required to produce facilitation of the test pulse MEP. The increased rTMS excitatory effects are possibly a result of inducing cooperativity as demonstrated in the early LTP paper by McNaughton (McNaughton et al., 1978). AP stimulation was found to be more excitatory than PA stimulation as demonstrated earlier in rTMS studies with 5 Hz rTMS (Rothkegel et al., 2010; Sommer et al., 2013a).

RMT decreased consistently with increasing pulse duration (Figure 3A) in agreement with previous reports (D’Ostilio et al., 2016), and the canonical strength–duration model of membrane excitation. The strength–duration time constants estimated for both PA and AP pulses (∼60–110 μs) were significantly below previous estimates (∼200–250 μs) with magnetically monophasic pulses (Peterchev et al., 2013; D’Ostilio et al., 2016). This discrepancy may be due to the different pulse shapes: we used magnetically biphasic pulses (*M* = 1), inducing more symmetric, triphasic E-field waveforms, which potentially recruits a mixture of neuronal populations (Sommer et al., 2018). Activation of a mixture of populations with different membrane time constants would alter the overall shape of the strength–duration curve, making more ambiguous the physiological meaning of the strength–duration time constant estimates. Nevertheless, we estimated higher time constants for AP pulses relative to PA, which agrees with the trend observed for time constants measured during voluntary contraction, but not at rest (D’Ostilio et al., 2016).

We also sought to explain how changing the pulse duration might alter the effects of 5 Hz pulse trains on MEP modulation, despite each pulse duration producing equivalent motor output at either 80 or 90% RMT. Any differences in plastic aftereffects of pulse trains must be related to differences in the single pulse effects. Using a simple low-pass filter to model membrane polarization, we found that peak depolarization correlated well with MEP modulation across all conditions (Figures 5B,C), specifically for neural elements depolarized by AP-directed E-field and for membrane time constants near 200 μs. Previous studies have found that 5 Hz rTMS most effectively facilitates MEPs using biphasic pulses with the dominant phase in the posterior direction (AP) (Sommer et al., 2013a), suggesting neural elements activated by AP-directed E-field are involved in the neuromodulatory aftereffects. The polarity of membrane polarization (i.e., depolarization vs. hyperpolarization) is determined primarily by the relative orientation of the local E-field and secondarily by electrotonic interactions between differentially polarized branches within axonal and dendritic arbors (Tranchina and Nicholson, 1986; Arlotti et al., 2012; Aberra et al., 2018). Models and *in vitro* evidence suggest downward (pia to white matter) E-fields tend to depolarize the soma and basal dendrites of pyramidal cells while hyperpolarizing their apical dendrites (Bikson et al., 2004; Radman et al., 2009; Aberra et al., 2018). While cortical axons and dendrites typically have main branches aligned to the cortical columns, they also possess several oblique and transverse branches spanning virtually all possible directions, adding considerable complexity to polarization distributions induced within a given neuron. Therefore, the relevant neural elements may be axons belonging to the “AP-sensitive” neurons activated by single supra-threshold pulses with AP-directed E-fields, or dendritic elements oriented posteriorly, e.g., basal dendrites of neurons on the anterior side of the precentral gyrus or apical dendrites of neurons on the posterior side of the precentral gyrus (Sommer et al., 2013a).

The correlation between peak depolarization and MEP modulation was highest for model time constants between 0.1 and 1 ms (Figure 5C), while the estimated time constants from the motor threshold measurements (Figure 3A) were at the lower border of this range, suggesting the neural elements involved in producing the facilitatory effects at longer pulse durations were different from the directly activated elements. Additionally, since the pulse intensity is scaled to produce equivalent motor output, the directly activated elements producing corticospinal output likely experienced similar peak depolarization across pulse durations. Dendritic membrane time constants measured with intracellular electrodes are in the 1–10 ms range (Ranck, 1975), although time constants are known to be lower for extracellular stimulation, depend on the field distribution (Ranck, 1975; Radman et al., 2009; Rattay et al., 2012), and respond preferentially to wider pulses (Rattay et al., 2012). Therefore, this simple model suggests the facilitatory effects were dependent on polarization of AP-sensitive neural elements with time constants longer than the neural elements responsible for the TMS-evoked motor output (pyramidal tract activation). While speculative, if these longer time constant neural elements are dendritic membranes, the increased MEP facilitation may correlate with increased recruitment of dendritic plasticity mechanisms.

This scenario is similar to what McNaughton and colleagues observed in rat cortices, where they demonstrated cooperativity of multiple afferents brought about by higher intensity and wider pulses as a mechanism of LTP (McNaughton et al., 1978). We propose that cooperativity is probably mediated by a stronger and wider dendritic activation, as a result of the unique membrane properties of dendrites and their important role in inducing synaptic plasticity. However, in a previous study with a 5 Hz biphasic (AP) rTMS protocol facilitation was not blocked by the glutamate receptor antagonist dextromethorphan; this led to the conclusion in the discussion that the mechanism was post-tetanic potentiation, which is NMDAr-independent (Sommer et al., 2013b). So another possibility is that the effect of pulse duration could be mediated by differential polarization of the presynaptic terminals activated by each pulse (Habets and Borst, 2006). Current injection into dendrites furthest from the soma produced longer and larger action potentials compared with somatic current injection (Larkum et al., 2007), especially in response to stimulation with higher frequency and longer pulse duration (Ledergerber and Larkum, 2010).

Dendritic stimulation can also generate back-propagating potentials that potentiate the anterograde potentials arising from somatic stimulation, thus producing LTP through associativity (Larkum, 2004), or cooperativity and spike-timing dependent plasticity (Lenz et al., 2015). The significance of dendritic activation in producing lasting plastic aftereffects through LTP in response to rTMS has been emphasized in *in vitro* studies (Sjöström et al., 2008; Müller-Dahlhaus and Vlachos, 2013). The large dendritic capacitance is mediated by the dendritic surface area, a significant portion of which is contributed by dendritic spines. Dendritic spines can also passively amplify local synaptic depolarization up to 50-fold due to their higher input resistance and increase cooperativity due to the high spine neck resistance (Harnett et al., 2012). Dendritic firing requires longer rTMS trains, but when finally achieved, the firing has significantly larger amplitudes and lasts for a longer period after stimulation ceases (Lee and Fried, 2017). If the dendritic activation hypothesis is verified, the therapeutic efficacy of high frequency rTMS in treating neurological or psychiatric diseases (Lefaucheur et al., 2020) might benefit from manipulating the duration of the individual pulses to modulate the extent of dendritic activation.

This study has several notable limitations. Absence of 90% PA conditions was a limitation that did not allow for a free comparison of the direction and intensity effects. Moreover, within the 90% AP condition, the effect of the pulse duration on the MEP amplitude change after rTMS was indicated by exploratory *post hoc* analyses in the absence of a significant ANOVA effect. A factor contributing to the small effect size may be the narrow range of pulse durations (80–120 μs), as compared to our previous study with 1 Hz rTMS, which explored a wider duration range (40–120 μs) as well as both bidirectional and unidirectional pulses (Halawa et al., 2019). Therefore, future studies could seek confirmation and enhancement of our exploratory findings, potentially by refining the experimental paradigm to include a wider range of pulse durations and directionality conditions. Finally, we used a very simple neural membrane response model. In the future, more realistic neuronal representations embedded in 3D models of the individual head and brain could be deployed to better understand the mechanisms underlying the effects of various TMS parameters (Aberra et al., 2020).

## Data Availability Statement

The raw data supporting the conclusions of this article will be made available by the authors, without undue reservation.

## Ethics Statement

The studies involving human participants were reviewed and approved by the Local Ethics Committee of the University Medical Center Göttingen. The patients/participants provided their written informed consent to participate in this study.

## Author Contributions

IH, KR, MS, and WP conceived the research idea. IH and KR collected and analyzed the experimental data and prepared the initial figures and manuscript. AA and AP produced the modeling data. IH, KR, AA, MS, AP, and WP revised the manuscript and approved the final version. All authors contributed to the article and approved the submitted version.

## Author Disclaimer

The content is solely the responsibility of the authors and does not necessarily represent the official views of the funding agencies.

## Conflict of Interest

AP is an inventor on patents and patent applications on TMS technology, related to TMS, he has received travel support as well as patent royalties from Rogue Research for cTMS, research and travel support, consulting fees, as well as equipment donations from Tal Medical/Neurex, patent application and research support as well as hardware donations from Magstim, equipment loans and hardware donations from MagVenture, and consulting fees from Neuronetics, BTL Industries, and Advise Connect Inspire. The remaining authors declare that the research was conducted in the absence of any commercial or financial relationships that could be construed as a potential conflict of interest.

## Publisher’s Note

All claims expressed in this article are solely those of the authors and do not necessarily represent those of their affiliated organizations, or those of the publisher, the editors and the reviewers. Any product that may be evaluated in this article, or claim that may be made by its manufacturer, is not guaranteed or endorsed by the publisher.
