# Supplementary material for: Effect of Pulse Duration and Direction on Plasticity Induced by 5 Hz Repetitive Transcranial Magnetic Stimulation in Correlation With Neuronal Depolarization
Source: Front Neurosci. 2021 Nov 26;15:773792. doi: 10.3389/fnins.2021.773792 (PMC8661453; doi:10.3389/fnins.2021.773792)
Supplement: Supplementary file 1 [file Data_Sheet_1.PDF]

	80% PA	80			100			120		
		RMT [%MS	cTMS RMT	1mV	RMT [%MS	cTMS RMT	1mV	RMT [%MS	cTMS RMT	1mV
S1	1	39	25	45	41	24	48	42	21	50
S2	2	27	19	31	26	18	30	28	15	31
S3	3	25	15	30	25	15	30	27	14	32
S4	4	28	22	38	28	17	34	28	15	38
S5	5	45	31	53	51	28	56	51	28	58
S6	6	40	28	46	38	24	46	41	22	47
S7	7	40	32	48	45	30	56	38	19	50
S8	8	38	29	47	40	25	46	41	23	49
S9	9	31	24	37	33	20	36	33	19	40
S10	10	35	23	40	34	21	44	34	19	41
S11	11	34	26	40	38	23.5	45	33	18.5	40
S12	12	38	27	57	38	22	55	39	21	47
S13	13	33	23	40	33	20	39	34	17	40
S14	14	35	26	41	34	23	41	36	21	40

	80% AP	80			100			120		
		RMT [%MS	cTMS RMT	1mV	RMT [%MS	cTMS RMT	1mV	RMT [%MS	cTMS RMT	1mV
S1	1	40	33	48	39	25	43	38	24	43
S2	2	28	24	33	26	22	31	27	19	32
S3	3	25	23	31	26	21	31	26	20	31
S4	4	26	25	36	29	24	41	28	20	42
S5	5	43	35	52	42	30	50	43	29	52
S6	6	44	36	50	40	31	47	40	28	47
S7	7	39	30	48	40	26	52	36	24	50
S8	8	40	32	47	40	31	46	36	27	45
S9	9	32	30	38	33	25	36	33	19	36
S10	10	34	29	41	35	24	42	33	23	44
S11	11	34	30	41	36	29	43	33	25	39
S12	12	39	32	47	38	32	48	38	30	57
S13	13	32	28	39	33	25	39	34	24	41
S14	14	34	32	41	33	27	40	35	24	41

	90% AP	80						120		
		RMT [%MS	cTMS RMT	1mV	RMT [%MS	cTMS RMT	1mV	RMT [%MS	cTMS RMT	1mV
S1	1	33	29	37	33	25	36	33	20	35
S2	2	27	23	30	26	22	32	27	18	31
S3	3	26	24	31	26	21	31	27	20	31
S4	4	26	25	39	27	23	41	28	20	42
S5	5	40	34	59	41	29	52	35	26	54
S6	6	32	29	39	42	26	48	35	23	41
S7	7	38	31	46	39	27	48	38	25	47
S8	8	41	33	46	40	30	47	39	27	46
S9	9	32	27	39	33	25	36	34	21	41
S10	10	30	28	39	35	24	41	31	21	40
S11	11	39	36	46	36	31	45	42	28	50
S12	12	40	34	46	38	32	47	42	29	46
S13	13	40	36	54	33	28	45	39	24	54
S14	14	26	27	36	33	25	38	29	24	41
