# Supplementary material for: Effect of Pulse Duration and Direction on Plasticity Induced by 5 Hz Repetitive Transcranial Magnetic Stimulation in Correlation With Neuronal Depolarization
Source: Front Neurosci. 2021 Nov 26;15:773792. doi: 10.3389/fnins.2021.773792 (PMC8661453; doi:10.3389/fnins.2021.773792)
Supplement: Supplementary file 2 [file Data_Sheet_2.PDF]

PA

		S1	S2	S3	S4	S5	S6	S7	S8	S9	S10	S11	S12	S13	S14
80µs															
BL		1.85	1.35	1.37	0.35	0.77	2.14	0.70	0.83	0.94	0.90	1.37	1.01	1.00	0.80
	1.00	0.41	0.62	1.33	0.23	1.59	0.83	1.18	1.74	1.23	0.38	0.83	2.86	1.01	1.16
	2.00	0.69	0.84	1.91	0.15	0.80	1.37	0.95	2.11	1.65	1.13	0.73	1.52	1.12	0.99
	3.00	1.56	0.83	1.60	0.28	0.88	0.65	0.78	1.97	1.00	0.99	0.68	1.26	2.53	0.80
	4.00	1.89	0.89	1.79	0.23	0.69	0.80	0.79	1.78	2.00	0.45	0.85	1.18	1.11	0.79
	5.00	1.26	1.32	1.28	0.24	0.75	1.25	0.85	1.69	1.21	0.53	1.38	1.39	2.05	1.40
0min		1.71	1.08	1.39	0.16	0.95	1.56	0.69	2.07	0.97	0.57	0.94	1.55	0.53	0.64
5min		1.33	1.04	1.92	0.27	0.83	0.82	1.39	2.49	1.21	0.29	1.00	1.41	0.92	0.38
10min		1.56	1.12	2.44	0.38	0.90	1.29	0.81	2.45	1.42	0.66	1.58	1.61	1.05	0.59
15min		0.93	0.90	2.01	0.43	1.09	1.20	1.04	2.64	1.30	0.62	1.58	1.65	1.24	0.77
20min		1.42	0.96	1.89	0.35	0.14	0.67	1.04	2.35	1.14	0.68	1.12	1.30	0.80	0.53
25min		1.22	0.99	1.75	0.20	0.49	1.66	1.26	1.94	1.50	0.80	1.34	1.19	1.13	0.58
30min		1.09	0.84	1.97	0.33	0.54	1.81	1.34	1.96	1.36	0.88	0.97	1.24	1.48	0.51
100µs															
BL		0.97	1.46	0.72	0.52	0.99	1.34	0.66	1.22	1.46	0.79	1.20	1.58	0.67	1.83
	1.00	1.16	1.45	0.86	0.38	1.00	1.55	0.71	2.17	1.12	1.05	0.74	1.87	0.77	1.32
	2.00	0.79	1.29	0.58	0.41	1.07	0.91	0.72	2.72	1.19	1.27	0.73	1.69	0.75	1.29
	3.00	0.79	1.91	0.74	0.60	1.18	1.71	1.17	1.07	1.03	1.03	1.00	1.49	1.11	1.68
	4.00	0.61	0.77	0.81	0.75	0.51	1.51	0.48	2.23	1.10	0.89	0.87	1.91	0.89	0.95
	5.00	0.99	0.81	0.63	0.54	0.75	0.75	0.76	1.75	1.31	1.30	0.78	1.77	0.94	0.93
0min		1.38	1.36	0.30	0.54	1.02	1.26	0.98	1.68	1.35	1.39	1.26	1.47	0.63	1.36
5min		0.65	1.08	0.64	0.60	0.83	0.77	0.75	1.79	0.82	1.39	1.13	1.35	1.36	1.37
10min		1.16	0.83	0.58	0.73	0.36	1.03	0.94	1.63	1.02	1.03	0.85	2.04	1.36	1.83
15min		0.45	1.28	0.40	0.71	0.52	0.84	0.85	1.78	1.18	1.27	1.17	1.91	0.89	2.27
20min		0.77	1.01	0.35	0.76	0.77	0.74	0.95	1.82	1.07	1.43	0.96	1.89	1.38	3.13
25min		0.98	1.14	0.35	0.69	0.90	1.62	1.00	1.00	1.09	1.20	1.06	1.60	1.01	1.34
30min		0.76	0.97	0.31	0.73	0.63	1.17	1.15	1.46	1.34	1.18	1.13	1.03	0.91	2.77
120µs															
BL1		1.28	2.02	1.02	0.84	0.96	1.41	0.69	0.49	1.11	1.04	1.63	0.95	1.03	1.66
	1.00	0.70	1.14	1.44	0.87	0.75	0.79	0.57	0.50	0.57	0.71	1.11	0.73	0.90	1.05
	2.00	2.05	1.48	1.13	1.37	0.63	0.73	0.80	0.40	0.89	0.70	2.84	1.31	0.83	2.12
	3.00	1.59	1.77	0.90	0.94	1.29	1.57	1.11	0.45	0.68	0.69	1.74	1.22	1.49	1.13
	4.00	2.01	0.80	0.87	0.84	0.68	1.41	0.70	0.44	0.73	0.55	2.07	1.02	0.84	1.23
	5.00	1.93	1.57	0.77	0.65	0.67	2.03	0.68	0.63	0.52	0.41	1.80	1.01	1.04	0.84
0min		3.05	1.24	0.76	0.71	0.79	0.80	0.49	0.49	0.75	0.90	1.55	0.90	1.77	1.33
5min		1.96	1.48	0.78	0.70	0.67	0.98	0.40	0.63	0.78	1.10	1.99	1.04	1.83	0.93
10min		1.88	1.47	0.68	0.59	0.85	1.08	0.31	0.84	0.52	0.49	2.18	1.09	0.65	1.62
15min		2.11	1.52	0.84	1.05	1.05	1.01	0.41	0.91	0.45	0.76	1.93	1.35	0.97	1.60
20min		2.18	1.11	1.14	1.07	0.59	1.21	0.26	1.18	0.61	0.61	1.63	1.25	1.09	1.95
25min		2.88	1.55	0.64	1.15	0.43	0.43	0.37	1.28	0.52	0.59	0.80	1.68	0.88	0.99
30min		3.21	1.94	0.78	1.07	0.59	0.71	0.63	1.06	0.45	0.74	1.40	1.05	0.60	0.70

AP															
80µs															
BL		1.00	1.28	0.52	0.87	1.00	1.77	1.53	1.51	0.73	1.29	0.86	0.79	0.72	1.05
	1.00	0.81	1.24	0.61	0.74	0.85	2.45	1.32	1.20	0.89	1.43	0.79	0.79	1.50	0.87
	2.00	1.12	1.00	0.52	0.68	0.96	1.97	1.10	2.02	0.90	1.02	1.32	1.22	0.45	0.53
	3.00	1.10	0.79	0.66	0.68	0.95	2.98	1.08	1.99	0.59	0.81	2.04	0.90	1.06	0.90
	4.00	1.40	1.04	1.23	0.70	1.11	2.44	1.17	2.13	0.43	1.14	0.96	1.38	0.45	0.98
	5.00	0.85	0.64	0.72	0.55	0.84	4.12	1.16	1.84	0.98	0.96	1.39	1.41	0.63	0.78
0min		0.99	0.96	0.60	0.54	0.69	2.89	1.20	2.28	1.09	1.46	1.93	1.84	0.65	0.80
5min		0.80	1.21	1.05	0.52	1.09	2.33	1.42	3.05	0.86	1.38	0.96	1.20	0.43	2.01
10min		0.94	2.06	0.68	0.79	1.14	2.45	1.25	1.27	1.44	1.57	1.17	1.40	0.12	2.97
15min		1.32	1.14	0.36	1.03	1.05	3.08	1.43	2.85	1.33	2.06	1.35	0.88	0.17	3.62
20min		0.96	1.13	0.47	0.64	0.52	2.62	1.17	3.11	1.11	2.53	0.95	1.32	0.43	1.75
25min		0.78	1.18	0.80	0.66	0.71	2.04	1.18	3.28	1.01	1.08	1.29	1.22	0.37	1.46
30min		1.17	0.94	0.50	0.72	0.84	2.25	1.29	2.76	0.99	0.72	1.56	1.20	0.94	2.73
100µs															
BL		0.72	0.51	0.86	0.77	0.85	2.09	1.49	0.60	1.65	1.21	1.27	0.78	1.74	1.12
	1.00	0.98	0.79	0.58	0.99	0.59	1.75	1.48	0.49	1.37	1.28	0.87	1.34	0.59	1.07
	2.00	0.89	0.61	0.88	0.79	0.91	2.95	1.61	0.64	0.80	1.11	0.56	1.50	1.54	0.78
	3.00	1.19	0.52	0.98	0.96	0.89	2.97	2.46	0.49	1.43	0.94	0.65	1.04	1.63	0.61
	4.00	0.54	0.59	0.80	1.04	0.43	2.19	2.24	0.85	1.20	1.22	1.29	1.36	2.48	0.43
	5.00	0.86	1.29	0.98	0.66	0.73	2.75	1.88	1.11	1.17	1.47	0.74	1.28	1.35	0.91
0min		0.77	1.57	0.72	0.97	0.63	2.71	1.60	1.17	1.10	1.75	0.83	1.68	1.55	1.57
5min		0.93	1.28	0.73	1.09	0.90	3.14	1.79	0.74	0.57	1.50	0.59	1.72	1.49	1.05
10min		0.53	0.91	0.54	0.97	0.53	2.09	1.89	1.26	1.33	1.71	1.09	1.39	0.78	1.30
15min		0.72	0.53	0.59	0.72	0.43	1.74	1.61	0.70	1.40	2.21	0.80	2.26	0.52	1.24
20min		1.00	0.69	0.75	0.57	0.36	2.23	1.89	1.39	0.97	1.95	0.85	1.32	0.55	1.75
25min		1.25	0.68	0.56	0.72	0.62	2.14	1.71	1.30	0.86	1.78	0.62	1.40	0.71	1.28
30min		0.60	0.66	0.46	0.84	0.69	1.58	1.42	1.28	0.64	2.05	1.05	1.16	0.92	1.70
120µs															
BL		1.74	1.46	0.78	0.93	0.88	1.39	1.70	1.65	0.97	1.44	1.73	1.32	0.69	1.13
	1.00	1.39	0.82	0.74	0.90	0.79	0.88	2.77	1.37	1.35	1.50	0.94	0.93	1.89	0.87
	2.00	1.28	1.19	1.13	0.78	0.80	1.22	2.51	2.21	1.31	1.38	1.11	0.85	1.43	1.10
	3.00	0.85	0.89	0.87	0.72	1.00	0.85	2.01	2.51	1.04	1.69	1.29	1.05	1.88	1.15
	4.00	0.62	1.73	1.08	0.88	1.00	1.08	1.90	1.59	0.93	1.37	1.34	1.10	1.86	1.17
	5.00	0.97	0.59	1.01	0.89	0.81	0.81	2.80	2.14	1.08	1.33	1.51	0.42	0.80	1.63
0min		1.35	0.86	0.88	1.07	0.63	1.49	3.55	1.90	1.06	0.98	0.83	0.84	0.74	1.13
5min		0.72	1.12	1.10	1.17	0.63	1.29	2.14	2.19	0.94	1.31	1.08	0.84	1.29	2.76
10min		0.92	1.33	1.22	1.02	0.50	1.61	2.22	1.72	0.93	1.22	1.58	0.89	0.84	2.13
15min		1.02	1.01	0.95	0.88	0.58	1.41	2.54	2.49	0.78	1.29	1.08	0.65	1.40	3.18
20min		0.85	0.92	1.14	0.67	0.44	1.19	2.26	2.22	1.14	1.59	1.31	0.55	1.15	2.68
25min		0.54	0.85	1.47	0.70	0.62	1.43	2.91	2.69	0.92	1.33	1.29	0.58	2.03	2.87
30min		0.58	1.06	0.76	1.17	0.53	0.68	2.42	2.50	1.26	1.90	1.05	0.47	1.51	1.14

90% RMT		S1	S2	S3	S4	S5	S6	S7	S8	S9	S10	S11	S12	S13	S14
80μs AP															
Bl		0.90	1.24	0.74	0.75	0.86	1.22	0.96	1.24	1.34	1.43	1.30	0.90	1.05	1.29
	1.00	1.09	0.76	0.70	0.89	0.86	1.75	1.31	1.61	1.04	2.13	1.63	1.03	1.01	1.42
	2.00	1.00	1.53	0.70	0.97	0.67	1.67	2.11	1.79	1.30	2.70	0.72	1.51	1.56	1.23
	3.00	0.56	1.09	1.07	0.78	0.43	1.62	2.32	1.30	1.43	2.41	0.78	1.39	1.46	1.04
	4.00	0.39	0.93	0.61	0.72	0.65	1.84	1.59	1.33	1.01	1.65	1.45	1.34	1.48	1.06
	5.00	0.60	1.79	0.67	1.05	0.71	2.02	1.81	1.56	1.44	2.17	1.01	0.79	1.79	1.40
0min		0.67	1.80	0.37	0.91	0.75	1.63	2.93	1.54	1.02	2.22	1.38	1.78	1.26	1.00
5min		1.06	1.12	0.62	0.88	0.95	2.24	2.53	1.18	0.84	2.02	1.09	0.51	1.27	1.66
10min		0.70	1.61	0.58	1.23	0.94	2.23	1.73	1.86	1.01	2.03	1.27	0.94	1.11	2.14
15min		1.00	1.39	0.51	0.60	0.53	1.29	1.62	1.71	0.94	1.80	1.35	1.21	1.53	2.62
20min		0.99	0.98	0.79	0.87	0.55	1.74	1.42	1.65	1.12	2.03	1.26	1.07	0.82	2.89
25min		1.53	2.29	0.87	0.99	0.91	0.85	2.82	1.75	0.95	2.51	1.81	0.77	1.29	2.99
30min		2.00	1.96	0.74	0.79	0.43	1.61	1.07	1.75	1.12	2.27	1.81	0.93	1.49	3.13
100μs AP															
Bl		0.91	1.27	1.03	0.65	0.54	1.23	0.78	1.27	0.96	1.12	1.26	0.73	1.48	0.60
	1.00	1.39	1.27	2.30	0.45	0.46	1.38	0.88	0.83	0.82	1.07	1.42	0.86	0.56	0.70
	2.00	1.15	2.05	0.99	0.34	0.47	1.01	1.14	1.27	0.95	1.09	0.88	1.07	1.18	0.36
	3.00	2.04	1.56	1.02	0.48	0.55	1.64	0.89	0.59	0.91	1.62	2.01	0.73	1.28	0.42
	4.00	1.18	2.20	1.12	0.56	0.79	1.10	0.64	0.84	0.95	1.30	1.22	0.73	1.82	0.78
	5.00	0.52	1.03	1.62	0.38	0.43	1.00	0.80	1.40	1.12	1.28	1.65	0.87	0.75	1.76
0min		1.11	0.89	1.18	0.44	0.53	1.44	1.07	1.08	0.90	1.29	1.39	1.04	0.96	0.85
5min		1.64	1.78	2.01	0.71	0.63	1.43	0.64	0.71	1.92	1.45	1.87	1.36	1.43	1.05
10min		0.29	2.08	1.64	0.56	0.60	0.75	0.95	0.55	1.81	0.74	1.43	0.97	0.71	2.19
15min		0.77	1.86	2.07	0.49	0.57	0.76	0.67	1.50	2.10	1.59	1.50	1.46	1.43	2.54
20min		0.63	1.57	1.13	0.66	0.64	1.25	0.61	1.53	1.96	1.13	1.54	1.04	1.20	2.16
25min		1.39	2.02	2.35	0.48	0.41	1.43	0.84	0.84	1.79	0.97	1.76	1.25	0.84	1.57
30min		1.62	2.15	1.87	0.58	0.32	1.38	1.33	1.22	1.95	1.16	1.42	1.32	1.84	1.46
120μs AP															
Bl		1.61	0.98	0.81	1.02	0.99	0.82	1.03	1.10	0.98	0.97	1.02	0.86	0.77	1.27
	1.00	1.04	0.78	0.98	0.43	0.60	1.14	1.17	1.08	1.01	1.45	0.78	1.21	2.41	1.42
	2.00	1.02	1.29	0.73	0.60	0.73	0.87	2.17	1.65	1.22	1.53	0.76	1.43	0.22	1.16
	3.00	1.32	0.64	0.83	0.73	0.69	1.40	3.44	1.82	1.25	1.10	0.98	1.28	0.84	0.61
	4.00	2.17	0.75	1.04	0.53	0.71	1.63	2.39	2.06	1.24	1.71	0.75	1.25	2.37	2.21
	5.00	2.19	0.88	0.69	0.92	0.97	0.99	2.49	2.12	1.26	1.90	0.83	1.40	1.07	1.36
0min		2.28	0.49	0.96	0.46	1.09	1.49	1.97	2.44	1.01	1.93	1.18	1.21	1.45	2.43
5min		1.29	1.13	1.24	1.31	0.86	1.46	1.77	1.35	0.98	2.14	0.86	1.12	2.24	2.96
10min		0.98	1.04	0.96	0.85	0.90	0.71	1.61	1.27	1.31	0.91	0.96	1.43	2.10	3.39
15min		1.13	0.87	0.89	1.52	0.91	1.13	1.51	1.93	1.04	1.49	0.94	1.30	2.06	2.17
20min		0.93	0.69	0.97	1.62	0.97	0.29	2.58	2.52	1.28	1.14	0.90	1.27	1.25	2.73
25min		0.83	1.15	0.84	1.31	1.01	0.28	1.86	2.68	1.00	1.26	1.44	1.51	1.98	2.33
30min		0.97	1.01	0.84	1.36	1.35	0.42	2.70	2.47	1.43	1.73	1.26	1.80	1.21	3.12
